# Old Habits Die Hard? Lingering Son Preference in an Era of Normalizing Sex Ratios at Birth in South Korea

**DOI:** 10.1007/s11113-016-9405-1

**Published:** 2016-07-15

**Authors:** Sam Hyun Yoo, Sarah R. Hayford, Victor Agadjanian

**Affiliations:** 1 International Institute for Applied Systems Analysis, Schlossplatz 1, A-2361 Laxenburg, Austria; 2 Department of Sociology, Ohio State University, Columbus, OH 43210 USA; 3 Department of Sociology, University of Kansas, Lawrence, KS 66045 USA; 4 Wittgenstein Centre for Demography and Global Human Capital, Welthandelsplatz 2 / Level 2, 1020 Vienna, Austria

**Keywords:** Son preference, Sex ratio at birth, Fertility intention, Low fertility, Korea

## Abstract

South Korea was among the first countries to report both an abnormally high sex ratio at birth (SRB) and its subsequent normalization. We examine the role of son preference in driving fertility intentions during a period of declining SRB and consider the contribution of individual characteristics and broader social context to explaining changes in intentions. We employ data from the National Survey on Fertility, Family Health and Welfare that span 1991–2012. We find that reported son preference declined to a great extent but remained substantial by the end of the observation period, and that the intention to have a third child still differed by sex of existing children. Change in individual-level factors does not explain the decline in son preference, suggesting that broad social changes were also important. This study provides a better understanding of how son preference evolves in the post-transitional context of very low fertility.

## Introduction

In contexts with substantial gender inequality in adult roles, parents may have strong preferences for male over female children, expressed through gender differences in infant care and feeding, unequal allocation of health care, and prioritization of sons’ over daughters’ schooling (see, e.g., DeRose et al. 2000; Lloyd 2005; Mishra et al. 2004; Yount 2003). Where voluntary fertility limitation is practiced, parents may also make decisions about when to stop childbearing based on the desire for male children, resulting in skewed sex ratios at last birth (SRLB) and different sibship patterns for boy and girl children (Bongaarts 2013; Gu and Roy 1995; Park 1983). Starting in the 1980s, as technology became available to allow sex-selective abortion, some countries in East Asia began to report sex ratios at birth (SRB) substantially higher than the expected level of around 105 male births per 100 female births (Guilmoto 2009). This manifestation of son preference has now been observed across East, South, and Central Asia (Attané and Guilmoto 2007; Bongaarts 2013; Das Gupta and Mari Bhat 1997; Duthé et al. 2012; Guilmoto 2012, 2015; Park and Cho 1995; Retherford and Roy 2003).

The expression of son preference changes over the transition from high to replacement-level fertility rates, and skewed SRBs are expected to decline in the post-transitional stage (Bongaarts 2013). However, the process and pace through which these ratios might return to normal levels is not well understood, and there is limited empirical evidence on the degree to which underlying preferences for sons may persist in very low-fertility contexts. Given the complex interactions between gender systems, individual attitudes, and demographic constraints that produce skewed SRBs, the factors shaping their normalization are likely to be equally complex.

In this article, we use data from repeated cross-sectional surveys to examine changes in son preference and fertility intentions during the period of falling SRB in South Korea, one of the first countries in the world to experience both increases in the SRB and a return to normal levels. We account for changes in men’s and women’s education and labor force participation during this period and speculate on the possible implications of changes in family law and family roles. Results show that, despite stabilization in the SRB, women continue to express a preference for sons, and intentions for second and third children are still dependent on the sex of children that they already have. Contrary to some assumptions that gender preferences for children have little impact in contemporary low-fertility contexts (e.g., Morgan 2003), our research provides evidence that son preference continues to be an important factor in understanding fertility intentions even in contexts of very low fertility with normal SRBs.

## Son Preference in Low-Fertility Contexts

Son preference has been observed in many parts of the world, but most commonly in South, Central, and East Asia (e.g., Andersson et al. 2006; Attané and Guilmoto 2007; Bongaarts 2001, 2013; Das Gupta and Mari Bhat 1997; Guilmoto 2009, 2012, 2015; Dahl and Moretti 2008; Park and Cho 1995; Pollard and Morgan 2002; Retherford and Roy 2003). Although the exact nature of gender systems varies across countries where son preference is observed, in general kinship systems that include patriarchy significantly contribute to the persistence of son preference. Most countries with son preference in Asia have a patriarchal tradition based on agriculture. Under this type of system, the family line and family resources (e.g., land and property inheritance) are transmitted predominantly through male descendants, who also have responsibility for filial duties, such as elder support and rituals for ancestor worship. In such societies, women do not have power and are marginalized, resulting in strong discrimination against women. Having sons also provides an economic advantage over having daughters: sons contribute more to economic production and provide old age support in an agricultural society, whereas daughters often become an economic burden due to the high cost of marriage.

The general preference for sons can be expressed through the treatment given to children, for example in the allocation of food, schooling, health care, and other resources, or through reproductive decision-making. Guilmoto (2009) identified three conditions that are necessary for abnormally high SRBs: a preference for sons that is strong enough to motivate sex selection, low fertility that generates “fertility squeeze,” and access to sex-detection technology. In pre- and early transitional societies where families typically have many children and the probability of having one or two sons is high, fertility behaviors are not strongly influenced by son preference. When fertility declines, the desire for small families can come in conflict with particular preferences for the sex composition of children, thereby creating “fertility squeeze.” However, even where the demand to limit fertility is present, unbalanced SRBs are not possible unless sex-detection technology enables families to carry out their desire to have sons rather than daughters. For example, Japan, a society where traditional gender ideology also favored sons over daughters, never experienced unbalanced SRBs because the transition from high to low fertility was largely completed before the introduction of sex-detection technology.

According to Bongaarts (2013), the demographic consequences of son preference change according to both the strength of preferences and the stage of the fertility transition. In the pre- or early transition stages, when son preference is the strongest, gender differences in infant and child mortality are often observed because son preference is realized through postnatal practices discriminating against girls. In the mid-transitional stage, when son preference remains high or declines slightly, SRBs and SRLBs begin to increase. As family planning (e.g., abortion, contraceptive use, and sterilization) expands, couples stop childbearing when they reach their desired numbers of boys (sex-specific stopping behavior). As a result, the youngest child is more likely to be a boy, leading to high SRLBs. However, the rise in SRLBs precedes that in SRBs due to limited access to abortion. In the late-transitional stage, when son preference remains strong even if it starts declining, both SRBs and SRLBs reach the highest levels. As the decline in desired number of children makes for “fertility squeeze,” couples continue to use sex-specific stopping behaviors and rely on sex-selective abortion in places where it is available.

The relationship between son preference and demographic outcomes at different stages of fertility transition, however, leaves unanswered the question of how son preference and its implementation change in the post-transitional stage, presumably due to limited empirical evidence. In post-transitional societies, where the family has gotten smaller and kinship networks have weakened, economic advances and improvements in gender inequality can contribute to the decline in son preference (Chung and Das Gupta 2007). For instance, a rise in absolute income can mitigate individual concerns for economic security in later life and in turn, may reduce the value of children, particularly the value of sons, as a means of securing old age support (Edlund and Lee 2009). A decrease in the wage gap between men and women also reduces the differences in the relative value of sons over daughters (Lee 2013). Nonetheless, in today’s Korea, gender inequity, while diminishing, remains pervasive, and women are still responsible for the majority of child-raising, which greatly limits their professional opportunities (Ma 2013, 2014). Given that son preference has existed in Korea for centuries, it is unlikely to disappear completely in a decade or so. We propose that son preference may linger as an underlying outlook even as its behavioral expression changes.

In this article, we examine the evolution of preferences for sons in South Korea (hereafter, Korea) over the past two decades when SRBs returned to normal levels. We focus on attitudes and intentions in order to understand how underlying preferences evolved as behavior changed. During this period, women’s educational attainment increased, as did the number of women working for pay (Korea National Statistical Office 1999; Statistics Korea 2013). In addition, the government-passed policy measures aimed at reducing SRBs as well as making legal changes to reduce institutionalized son preference. We consider the relative importance of both individual characteristics and large-scale social change in explaining changing attitudes.

## Fertility Decline and Evolving Son Preference in Korea

Korea experienced one of the most rapid transitions from high to very low fertility. The country’s TFR, which was around 6.0 in the early 1960s, reached replacement level in the early 1980s and further declined to very low levels by the early 2000s. Since then, the TFR has remained relatively stable below 1.3. A preference for sons over daughters persisted throughout this period but was manifested in different ways. In the periods of high fertility, gender differences in infant and child mortality were observed, probably due to less care for and neglect of female children (Choe 1987; Choe and Kim 1998). For instance, when a newborn was female, Korean women tended to conceive the next child quickly in order to have a son, resulting in a shorter birth interval after a female birth (Nemeth and Bowling 1985; Rindfuss et al. 1982). During the early- and mid-transition in which fertility declined rapidly, SRLBs began to increase (Park 1983). Although desired family size fell rapidly, most couples still wanted to have at least one or two sons. As a result, most women were reluctant to stop childbearing until they reached the desired number of sons. In the 1970s, induced abortion was widely used to stop childbearing or to ensure birth spacing (Cho et al. 1982; Choe and Kim 1998; Hong and Tietze 1979; Hong and Watson 1972), but it was not sex-selective until the early 1980s when sex-detection technology became available. As a result, son preference led to higher fertility rates in this period (Arnold 1985; Larsen et al. 1998).

**Fig. 1:**
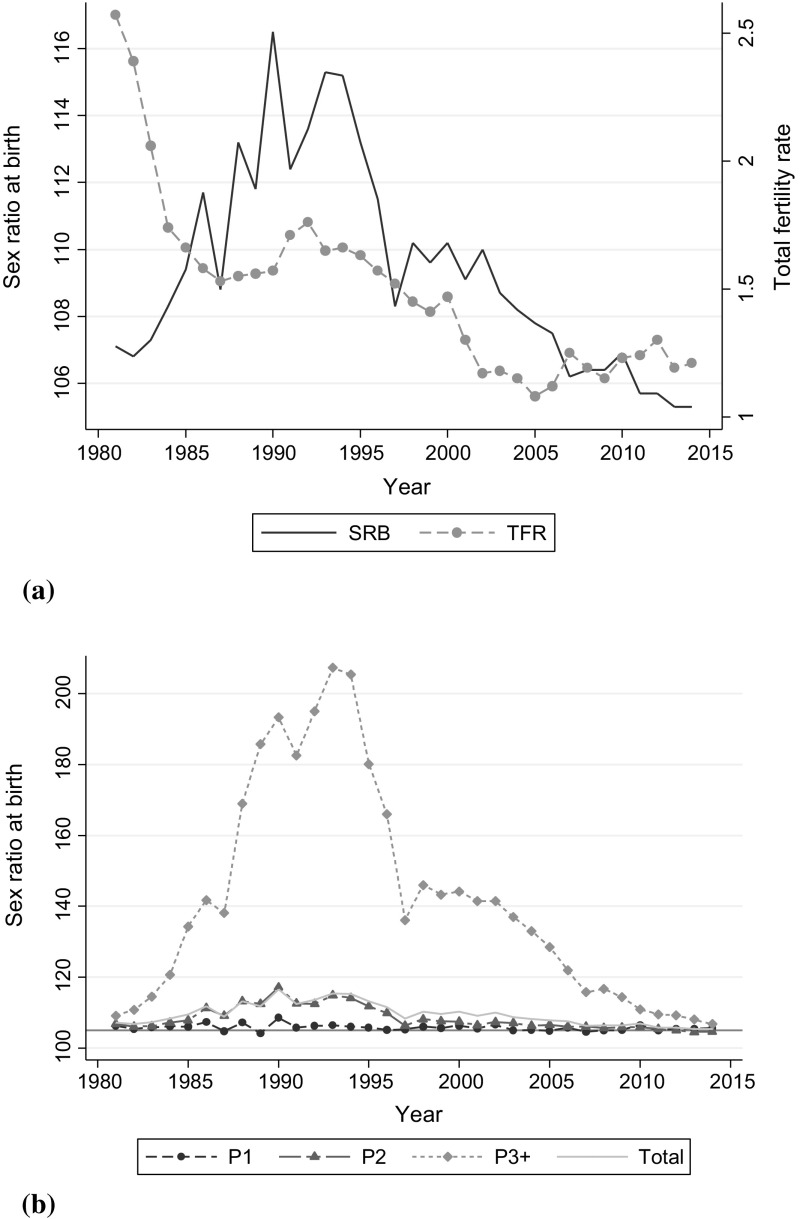
Total fertility rate and sex ratio at birth in Korea, 1981–2014. **a** Sex ratio at birth and total fertility rate. **b** Sex ratio at birth by birth parity. *Source* Statistics Korea (2015)

Figure 1 illustrates the joint trends in TFR and SRB between 1980 and 2012. In the beginning of the 1980s, when TFRs reached replacement level, SRBs began to depart from the normal level, 105 males over 100 females (Park and Cho 1995). The systematic rise in SRBs did not start until the early 1980s, when fetal sex detection became available. Since that time, induced abortion, which had been used for preventing unwanted births in general, began to be increasingly used for achieving sex preferences. While fertility rates continued to fall in the 1990s, SRBs peaked in 1990 at 116.5 male births per 100 female births. The SRBs gradually declined through the 1990s and returned to normal levels by the end of the 2000s. Since the early 2000s, overall SRB has continued to decline and reached an approximately normal level of 105.3 in 2013. In parallel, the attention to unbalanced SRB among policy makers gradually faded away. However, SRBs were still somewhat high for third- or higher-order births until recent years (108.0 males per 100 females in 2013) and for certain regions and groups (Kim 2011).

Induced abortion has been illegal in Korea since the 1950s, but because of the strong push for family planning, abortion laws were rarely enforced. As the unbalanced SRB became a serious social issue, in 1987 the Korean government adopted and enforced the Prohibition of Ascertaining the Sex of Fetus, a law that, as its name suggests, bans medical personnel from determining the sex of a fetus and notifying a pregnant woman, her family, or others. The law was modified in 2009 so that determining the sex of a fetus became possible only after 32 weeks of gestation.

Since 1990, aspects of the family law system that reinforced the traditional patrilineal kinship rules have gradually been modified and amended. For instance, the *hoju* system, which required all families to use the family name of the (male) head and prioritized the order of succession to the oldest son over other sons, daughters, and other relatives, was amended in 1990 and 2002 and finally abolished in 2005. These institutional changes occurred along with the expansion of women’s social and economic participation. For instance, women’s college enrollment rate rose from 22.5 % in 1980 to 65.4 % in 2000, and eventually reached 74.3 % in 2012, which is one of the highest levels in the world (Korea National Statistical Office 1999; Statistics Korea 2013). The Korean government also reversed its antinatalist population policy in 1996 and later launched the first (2006–2010) and second (2011–2015) Seromaji Plans (a combination of Korean words meaning ‘new beginning’ and ‘welcoming’) to raise fertility and to prepare for aging society (Lee 2009). These policies include improving gender equity and providing support for balancing work and childrearing.

Despite these improvements, gender roles still remain largely grounded in traditional norms. A wife typically carries out the majority of household chores, while a husband focuses on paid work. According to the 2009 Time Use Survey, in a Korean household of two parents with at least one child under age 18, on average women spent 4 hours and 53 minutes in household chores and taking care of family per day, while men spent only 1 hour and 43 minutes (Statistics Korea 2013). Traditional gender roles and corresponding stereotypes also continue to influence women’s social activities and economic participation. Korean young women actively participate in the labor market, but many of them exit it when they start childbearing and childrearing (Park and Kim 2003). Some of them re-enter employment much later, after raising children, but usually get low-skilled, low-paid, and low-status jobs due to the discontinuation of careers and gender-segmented labor market. Employment and motherhood are still viewed as competing for most Korean women, hindering their economic activities to a great extent (Ma 2013). As a result, female labor force participation rate (age 15+) has stalled around 50 % in the last two decades (Statistics Korea 2013). In societies where cultural change lags behind economic advancement, these lingering traditional gender roles may continue to influence decisions about bearing and raising sons and daughters. Due to this influence, a preference for sons may translate into sex-selective abortion or sex-based stopping patterns. It is also possible that dominant societal gender norms affect intentions for having another child even net of parents’ own gender preferences. For instance, if parents invest more time and resources in caring for sons than for daughters, or if boys are perceived as more difficult to raise than girls, parents of boys may be less likely to want another child than parents of girls (e.g., Choi and Hwang 2015; Dahl and Moretti 2008).

Previous research has related unbalanced sex ratios at birth in Korea to diverse factors, such as Confucian culture, health care access, migration, religion, residence, and other traditional norms like Chinese zodiac years (e.g., Kim 2004, 2005; Larsen et al. 1998; Lee and Paik 2006; Lee 2003; Park and Cho 1995). For instance, the skewed SRB has been more salient in the southeast region, the home of Korean Confucianism (Kim 2011; Kim and Song 2007; Kim and Lee 1999). It is also negatively associated with women’s education and urban residence (Chung and Das Gupta 2007, 2011; Kim 2011). Most studies, however, looked at individual and contextual factors from a cross-sectional perspective and thus, there is limited understanding of the nature of changing preferences over time.

Research on the decline in son preference emphasizes macrosocial changes. Early declines in son preference in Korea were primarily attributable to large-scale social change rather than increases in education or autonomy for individual women (Chung and Das Gupta 2007). Relative increase in women’s earnings potential also contributed to the declining trend in son preference (Lee 2013). However, Chung and Das Gupta (2007) only focused on the period between 1995 and 2003; also, they used self-reported son preference as the dependent variable, which may be biased by the existing sex composition of children (see our descriptive results below). The Lee (2013) study is based on aggregate-level analysis and does not account for individual characteristics. We build on this and other research by extending the period of study to cover the first decade of the century, in which the SRB returned to normal levels, and by analyzing fertility intentions as well as reported son preference at the individual level.

### Analytic Approach

Our analysis consists of two parts. First, we identify trends in several measures of son preference during the period 1991–2012. Despite the normalization of the SRB in this period, it is unclear whether the decline in SRB resulted from changes in underlying attitudes and preferences or whether it mainly reflected the increased state regulation of sex-detection technology. To understand it, we calculate the SRLB between 1991 and 2012 and present descriptive tables showing reported son preference and fertility intentions by sex composition of previous children.

Second, we assess the degree to which individual characteristics explain trends in sex-composition-specific fertility intentions. We estimate logistic regression models of the intention to have another child controlling for the sex of the previous child(ren). Our outcome measure does not identify whether a respondent specifically prefers to have a son or a daughter. However, aggregate-level differences in order-specific fertility intentions point to the existence of son preference at the societal level. We include interactions between sex of existing children and survey year to test whether the difference in intentions changed over time. Multivariate analyses also account for individual and household characteristics, such as both men’s and women’s employment and educational attainment, and ideal family size, as well as regional variation: we examine whether changes in these factors can explain changes over time in sex preferences by comparing Y-standardized coefficients across nested models.

## Data and Methods

### Data

The data for this study come from the National Survey on Fertility, Family Health & Welfare (NSFFHW) 1991, 1994, 1997, 2000, 2003, 2006, 2009, and 2012. The NSFFHW has been conducted by the Ministry of Health and Welfare in cooperation with the Korea Institute for Health and Social Affairs. The surveys have changed titles several times but are essentially a set of repeated cross-sectional surveys. Although the surveys have been continuously conducted since the 1970s as part of the World Fertility Survey, the survey data before the 1990s are not available, in part due to loss of microlevel data. Each survey is based on a nationally representative sample of both households and women of reproductive age. The surveys use three-stage stratified sampling based on census enumeration districts, but the sampling procedure varies by survey year. The sample size for each survey also varies, but it is usually around 10,000 households and women living in those households. Despite variation in survey designs, samples, and questionnaires, the survey maintains the standard primary questionnaires on pregnancy history and intended fertility.

We restrict our analytic sample to currently married women aged 15–44 (40,426 cases across all the surveys). In some surveys, previously married women are also included in the sample; we limit the sample to currently married women in all years to increase comparability (All surveys include ever-married women only. Such survey designs are not uncommon in Korea, where nonmarital births are still rare.). Because of continued increase in age at marriage in the past decades, the mean age of women in our sample also shifted from 33.6 to 36.5 between 1991 and 2012 (Table 5 in Appendix). Thus our analysis may confound change over time with changes in age composition. We control for age in multivariate models. In addition, to assess the degree of potential bias, in addition to a model that includes women of the entire age range, we also estimated models restricting the sample to women aged 25–34 and aged 30–39 separately, i.e., the age range when most childbearing occurs; results are substantively similar to those from the full age range (see Tables 6 and 7 in Appendix). We prefer the wider age range in order to include women who marry at unusually young or old ages. After excluding 351 women who are infertile (or living with an infertile husband) or have missing values on children ever born or fertility intentions, our final analytical sample includes 40,075 women. All cases are used for descriptive analysis. Because we conduct separate analyses by birth order, the sample size for multivariate analyses varies: 8686 women with one child and 22,008 women with two children.

The surveys provide weight variables for households and individuals (women) separately, but for the 2009 survey only the household weight variable is provided. According to the descriptive report of the 2009 survey (Kim et al. 2009), individual weights were computed by adjusting household weights based on individual (women’s) response rates in sampling units. Thus, the individual weight should not be very different from the household weight. Unfortunately, the response rates were not published either, and we were unable to calculate individual weights for the 2009 survey. Instead, we use household weights for this survey and individual weights for other surveys. In addition, as the scale of the weights varies somewhat with survey year, we normalize the weights by dividing the weight by its mean so that the mean of weights is equal to one for all surveys, while the sum of weights becomes the actual sample size for each survey.

### Measures and Models

We use several direct and indirect measures of son preference: the sex ratio at last birth (SRLB), reported son preference, and fertility intentions as a function of sex composition of existing children. The SRLB represents the indirect outcome of past son preference implementation, primarily through fertility stopping behaviors. Following Bongaarts (2013), we calculate the sex ratio of the most recent birth that occurred in the past 10 years among women who want no more children at the time of interview. The SRLBs are directly measured from the information on either respondent’s last live birth or pregnancy history, but this information is not available in the 1994 survey. Thus, we provide SRLBs for all survey years except 1994.

We also use two individual-level measures of son preference, one derived from a direct question on son preference and another based on whether the respondent wants another child. Each survey asks married women, “Is it necessary for you to have a son?” Possible responses include “must have a son,” “would be better to have a son than none,” “doesn’t matter,” and “don’t know.” We transformed responses to this question into three categories, “must have a son,” “better to have a son,” and “does not matter or not sure” (no son preference). We separate “must have a son” from “better to have a son” (except in some basic descriptive tables) because we are interested in differentiating between stronger and weaker preference for male children. We combine “don’t know” with “doesn’t matter” because the proportion of women reporting “don’t know” is less than 1 % of the analytic sample and because no regular pattern is observed from these cases. A similar measure was used in an earlier study describing the decline in son preference in Korea (Chung and Das Gupta 2007). It captures underlying feelings about the desire for sons and has been shown to affect fertility independently of other individual characteristics (Larsen et al. 1998).

Reported son preference may underestimate actual predilections if people are reluctant to admit their bias. At the same time, reported son preference may overestimate the influence of predilections on behavior if such predilections are not strong enough to act on or if sex-selective technology is not readily available. We therefore also include a measure that is more closely linked to behavior—the intention to have another child. Although fertility intentions do not perfectly predict actual fertility, they are among its strongest determinants (Schoen et al. 1999), serving as a link between underlying values, reproductive goals, and eventual behavior (Bachrach and Morgan 2013; Barber 2001). Our measure of fertility intentions comes from the question “Do you want to have another child?” Possible responses include “yes,” “no,” and “under consideration.” Pregnant women are asked about their intentions for another child beyond current pregnancy. We recoded it into a dichotomous variable, whether or not a respondent wants to have another child (1 = yes/under consideration, 0 = no), so that we can estimate the likelihood of wanting or considering another child. In exploratory analyses, we tested models dropping “under consideration” responses, combining these responses with the “no” rather than the “yes” category, and treating them as a separate response category. Results were substantively similar across all specifications.

We use logistic regression models to estimate the association of individual characteristics and time period with intentions to have another child. The core measure of son preference is whether fertility intentions differ by the sex composition of existing children. Because fertility rates in the time period we are studying are low, we do not examine women with more than three children; among women with three children, just 1.3 % expressed an intention to have another child in 2012. We incorporate sex composition of existing children into analytic models as a set of dummies, depending on the number of children. We group women with two children into three categories: mixed-sex (one son and one daughter), two sons, and two daughters. For women with only one child, we distinguish between whether the first child is a son or a daughter. Due to data limitations, we do not consider the birth order of sons and daughters for women with two children. We set up having one son and one daughter as the reference for women with two children and having a son for women with one child.

We test whether the sex composition of existing children affects fertility intentions for another child and how that effect may change over time by interacting survey year with sex composition, while taking into account period change in fertility intentions. We also consider several covariates that may affect both son preference and fertility intention and that changed substantially during the period of study. Following prior research, we include area of residence (metro, small city, and rural) in our model. We also include woman’s educational attainment (high school completion or less, some university, and bachelor’s degree or higher), employment status (not employed, white-collar worker, and blue-collar worker), and age (5-year age groups). Household income may also be associated with fertility intentions. However, in our data information on income is available only after the 2000 survey. Because male-breadwinner families are still dominant in Korea, household income is largely determined by men’s employment. Moreover, prior research suggests that the progression to second- or higher-order births has become increasingly selective on socioeconomic conditions, such as job security and occupational status, after the Asian financial crisis (Kim 2007, 2013; Yoo 2014). We therefore include husband’s educational attainment and employment status as proxies for household income. For these variables, we use the same categories as those used for women.

One of our primary interests is whether changes in individual characteristics, such as educational attainment, employment, ideal family size, and residence area, explain changes over time in sex preference. We compare coefficients between a baseline model and a full model with individual characteristics. As logistic regression does not allow us to directly compare coefficients across models, we use Y-standardized coefficients instead in order to compare results across models that have the same sample but different independent variables (Mood 2010). These coefficients account for changes across models in unexplained variance that produce changes in the scale of the coefficients. All models use robust standard errors to account for sampling designs.

## Results

### Descriptive Results

**Fig. 2:**
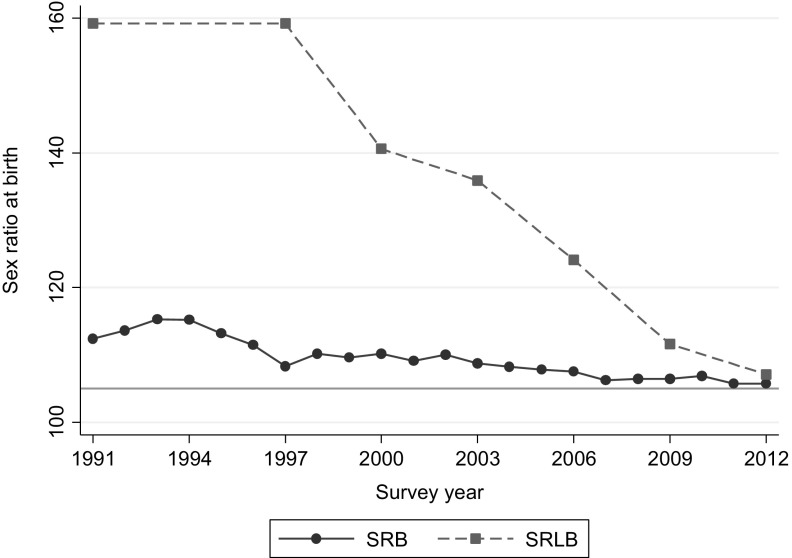
Sex ratio at birth and sex ratio at last birth. *Source* The SRB is from Statistics Korea (2015). SRLB was computed by authors from National Survey on Fertility, Family Health and Welfare, various years (1991–2012). Because of data limitations, SRLB is not available in 1994. Please see the text for details

Figure 2 shows trends in Korea’s SRLB. For comparison, the SRB is also included in the figure; the SRB is released by Statistics Korea every year based on vital registration data. As described in the introduction above, the SRB peaked in the early 1990s and declined steadily, reaching normal levels in 2012 (105.7 males per 100 females). The SRLB did not change until the late 1990s, but then rapidly declined, reaching 107.1 males per 100 females by the end of the study period. As the SRLB was at very high levels in the 1990s, its declining trend was steeper and faster than that of the SRB. Differences in trends are partly due to the differing time spans described by these measures: the SRB is based on births in a calendar year and immediately casts change in reproductive outcomes, while the SRLB uses the most recent births in the last 10 years among women who stopped childbearing (on average these births occurred about 4.6 years earlier in our analytic data) and thus involves more cumulative outcomes. Differences between SRB and SRLB also reflect the different determinants of these two measures. The SRB can only be elevated by sex-selective abortion, while the SRLB is sensitive to contraceptive use and stopping decisions. Nonetheless, the rapid drop of the SRLB from 159.2 to 107.1 between 1991 and 2012 is remarkable. Figure 2 also suggests that the decline in sex-selective abortion was followed by a decline of gender-based stopping pattern, albeit with a time lag. It implies that son preference was becoming increasingly less important in childbearing decisions.

Reported son preference and fertility intentions also demonstrate declining but still noticeable preference for sons. Table 1 presents reported son preference by birth order and sex composition of existing children for women in each survey year. Overall, the proportion of married women who reported either that it is necessary to have a son or that it is better to have a son fell from nearly 69.2 % in 1991 to only 40.5 % in 2012. Despite this rapid decline, the proportion of women reporting son preference is still substantial. Furthermore, in most years, the proportion was higher among women with two children than among those with one child. This association may be due to a direct relationship between son preference and family size norms, or it may be attributable to age or cohort effects, because women with two children are older on average than women with one child. In these data, women with two children are about 4 years older than women with one child, on average (see Table 5 in Appendix); thus, age differences are unlikely to fully explain this association.

**Table 1:** Percent of women expressing son preference (must have a son + better to have a son) by the number and sex composition of children. *Source* National Survey on Fertility, Family Health and Welfare, various years (1991–2012)

	No child	One child			Two children				Three or more	Total
	All	1 dau.	1 son	All	2 dau.	1 dau. + 1 son	2 sons	All	All	
1991	52.1	56.0	63.8	60.5	52.9	74.0	70.4	69.6	83.7	69.2
1994	44.6	51.1	49.0	49.9	50.1	66.7	63.4	62.9	76.8	60.4
1997	43.5	44.8	54.9	50.6	42.4	66.6	61.9	61.5	75.2	59.4
2000	41.9	42.0	53.9	48.8	45.7	67.7	62.6	62.5	72.0	58.5
2003	40.0	42.4	53.0	48.6	36.5	62.4	60.0	57.4	66.9	55.4
2006	42.7	36.5	49.1	43.6	36.6	53.9	51.1	50.0	59.6	49.0
2009	39.5	35.9	50.2	43.9	36.6	52.8	53.2	49.6	54.8	47.9
2012	35.1	26.0	46.1	36.2	26.8	45.0	45.4	41.5	51.4	40.5
Total	42.5	41.6	52.7	47.9	40.8	61.7	59.2	57.3	70.6	55.6

All percentages are weighted. Unweighted *N* = 40,075 women

Consistent with declining fertility rates during this time period, the proportion of women who wanted another child fell between 1991 and 2012, as shown in Table 2. Among women aged 15–44, the share of those who wanted to have another child was 25.1 % in 1991 but decreased to 22.5 % in 2012. Overall, the downward trend in fertility intentions is also observed in order-specific tabulations among women with one or two children despite some variation in recent years. Intentions to have another child significantly decline as birth order increases. However, the most important result is that having no son at any birth order is associated with higher fertility intentions than other sex compositions of children. For instance, in 1991 women who had two daughters and no sons expressed an intention for another child almost 9 times more often than did those with two sons (29.7 vs. 3.4 %). The gap decreased continuously in the following two decades. Women with two daughters remained more likely to want another child than women with two sons (5.6 vs. 3.2 %) but the difference is no longer significant in 2012 when fertility intentions decreased to very low levels. A similar pattern is also observed among women with one child: women with only a daughter (no son) wanted another child more often than those with only a son. Again, the difference declined during the period under study but was still present by 2012. There are two slight rises in the intention to have another child, in 1994 and 2006. These fluctuations are likely attributable to the influence of the Chinese zodiac on marriage and fertility, which has been described elsewhere (e.g., Lee and Paik 2006).

**Table 2:** Percent of intention to have another child by birth parity and sex composition. *Source* National Survey on Fertility, Family Health and Welfare, various years (1991–2012)

	No child	One child			Two children				Three or more	Total
	All	1 dau.	1 son	All	2 dau.	1 dau. + 1 son	2 sons	All	All	
1991	95.4	71.5	53.5	61.2	29.7	2.5	3.4	7.1	2.2	25.1
1994	92.3	74.3	60.5	66.4	29.7	4.8	7.9	10.0	3.5	29.3
1997	92.1	60.1	47.5	52.9	18.7	3.2	3.9	5.8	2.7	22.6
2000	88.6	55.1	43.3	48.3	12.9	2.7	3.4	4.6	1.6	21.9
2003	84.0	48.7	38.6	42.9	11.4	3.6	4.6	5.1	2.3	18.7
2006	89.2	49.1	41.6	44.9	12.4	4.2	5.0	5.9	3.2	21.8
2009	88.6	47.5	47.2	47.3	13.9	6.2	5.9	7.7	3.1	24.2
2012	88.8	44.7	36.5	40.6	5.6	4.0	3.2	4.1	1.2	22.5
Total	89.9	56.5	46.4	50.8	16.6	3.8	4.6	6.3	2.5	23.3

All percentages are weighted. Unweighted *N* = 40,075 women

Interestingly, Tables 1 and 2 show inconsistencies in reported intentions among women with no sons. Among women with two children, those with two daughters had the lowest percentages of reported son preference in Table 1, but they also showed the highest, even if gradually decreasing, intention to have another child in Table 2. This discordance is also observed among women with one child; women with a daughter demonstrated higher intention for another child, while they reported lower son preference than did those with a son.

There are three potential explanations for these inconsistencies. First, gendered family systems may affect fertility intentions net of individual desires for sons. For instance, women with daughters might be more likely to want another child than those with sons because the time and material costs for raising girls are lower than for raising boys (Dahl and Moretti 2008). Second, women with daughters only might underreport their preference for sons, considering and justifying the sex of their existing children. Third, it could be that the sex distribution of children is a result of son preference and the observed associations are due to reverse causality. In fact, the distribution of sons and daughters observed in the sample suggests that son preference already affected reproductive behavior. If we assume that the natural level of SRB is around 105 males per 100 females, about 48 % of women with only one child should have a daughter, and about 23 % of women with two children should have two daughters. However, in our sample the percentages of those having two daughters among women with two children rarely reaches these levels, supporting the reverse causality argument that women already implemented their son preference at parity one or two (see Table 8 in Appendix). When this possibility is accounted for, our results presented below, may underestimate the impact of son preference on intentions.

### Multivariate Results

In order to understand how changes in individual characteristics may have contributed to observed trends, we estimate multivariate models. In Table 3, we present coefficients from logistic regression models estimating the likelihood of wanting another child among women with one child based on the sex of the existing child, reported son preference, survey year, and individual characteristics. In Model 1, we only consider sex of the existing child, survey year, and the interaction between the two. In Model 2, we add the full set of covariates. Son preference is represented by the coefficients for having a daughter as well as the coefficients for the direct measure of reported son preference.

**Table 3:** Logistic regression of intentions to have another child on sex of child and other covariates, women with one child. *Source* National Survey on Fertility, Family Health and Welfare, various years (1991–2012)

	Model I			Model II		
	*b*	RSE	bStdY	*b*	RSE	bStdY
Sex of first child (ref: a son)						
A daughter	0.78	0.13***	0.42	0.80	0.15***	0.32
Interaction w/survey (ref: a son * 1991)						
A daughter * 1994	−0.13	0.19	−0.07	−0.05	0.21	−0.02
A daughter * 1997	−0.27	0.18	−0.15	−0.16	0.22	−0.06
A daughter * 2000	−0.30	0.18^†^	−0.16	−0.34	0.22	−0.14
A daughter * 2003	−0.38	0.18*	−0.21	−0.53	0.22*	−0.21
A daughter * 2006	−0.44	0.18*	−0.24	−0.38	0.22^†^	−0.15
A daughter * 2009	−0.76	0.19***	−0.41	−0.79	0.22***	−0.32
A daughter * 2012	−0.43	0.23^†^	−0.23	−0.44	0.28	−0.18
Stated son preference (ref: none)						
Better have a son				0.43	0.07***	0.18
Must have a son				0.61	0.09***	0.25
Ideal family size (ref: two children)						
Don’t know				−0.66	0.30*	−0.27
Less than two children				−1.55	0.09***	−0.63
More than three children				0.41	0.08***	0.17
Residence (ref: metro)						
Small city				0.01	0.07	0.01
Rural				0.24	0.08**	0.10
Woman’s education: (ref: ≤HS comp.)						
Some college				0.08	0.11	0.03
Bachelor or higher				0.09	0.12	0.04
Woman’s employment: (ref: not working)						
White-collar job				−0.15	0.09^†^	−0.06
Blue-collar job				−0.32	0.08***	−0.13
Husband’s education: (ref: ≤ HS comp.)						
Some college				0.36	0.11**	0.15
Bachelor or higher				0.36	0.13**	0.15
Husband’s employment: (ref: not working)						
White-collar job				−0.04	0.19	−0.02
Blue-collar job				0.07	0.19	0.03
Woman’s age (ref: <25)						
25–29				−0.17	0.14	−0.07
30–34				−1.00	0.14***	−0.41
35–39				−2.47	0.15***	−1.00
40–44				−3.92	0.18***	−1.59
Survey year (ref: 1991)						
1994	0.27	0.11*	0.15	0.20	0.13	0.08
1997	−0.24	0.11*	−0.13	−0.22	0.13	−0.09
2000	−0.41	0.11***	−0.22	−0.30	0.14*	−0.12
2003	−0.58	0.12***	−0.31	−0.24	0.14^†^	−0.10
2006	−0.48	0.11***	−0.26	−0.30	0.15*	−0.12
2009	−0.26	0.12*	−0.14	0.32	0.15*	0.13
2012	−0.70	0.16***	−0.37	−0.05	0.21	−0.02
Constant	0.14	0.08^†^		0.95	0.24***	
Log pseudolikelihood	−6065.39			−4499.58		
*N* (unweighted)	8686			8686		

Sample includes women with one child with no missing data on independent or dependent variables. Normalized weight was used. RSE denotes Robust Standard Errors and bStdY indicates Y-standardized coefficient

^†^ *p* < .10; * *p* < .05; ** *p* < .01; *** *p* < .001

In Model 1, the coefficient for having a daughter is positive and statistically significant, indicating that women with a daughter are more likely to want another child than women with a son in 1991, the reference year. The daughter–year interaction terms represent variation in this effect over time. The coefficients are all negative, indicating that the difference in fertility intentions between women with a daughter and women with a son declined over time; they are marginally significant starting in 2000 but increase in magnitude from year to year and become significant starting in 2003 through 2009 (at the *p* < .05 level). The total effect of having a daughter (main effect + the interaction term with survey year) indicates that intentions to have a second child did not vary according to the sex of first child since 2009. Interestingly, the total effect size of having a daughter was close to zero in 2009 (.78–.76 = .02), implying relative indifference in intentions according to the sex of previous child. This dip is probably attributable to the temporary rise in marriage and fertility between 2006 and 2008, a “double spring year” from early 2006 to early 2007 and the Year of the Red (Golden) Pig from early 2007 to early 2008. Many singles who had been delaying marriage got married and immediately had a child in this period. As this period was also auspicious for new born babies, these couple could have higher fertility intentions that are indifferent to child’s sex. This supposition is supported by the fact that fertility intentions in the 2009 survey year are higher than those in 2006 and 2012. As the introduction of pronatalist policies received much attention from mass media starting in 2006, there is also a possibility that women began to favor somewhat higher fertility in that period.

The full model that includes covariates is displayed in Model 2. Reported son preference is also positively and significantly associated with the desire to have another child. In Model 2, both thinking “better to have a son” and “must have a son” are associated with higher intention to have another child (.43 and .61, respectively, *p* < .001) when the sex of previous child, survey year, and the interaction between the two are controlled. The difference between “better to have a son” and “must have a son” was marginally significant (*p* < .10). Y-standardized coefficients for other covariates remain stable in models without the direct measure of reported son preference (not shown here), suggesting that this measure has an independent association with fertility intentions net of sex composition of children. The persistence of the association of sex composition of children with intentions after controlling for reported son preference implies that pressures to have a son influence fertility intentions even among women who do not explicitly state in a survey that they would prefer to have a son.

Women’s employment and husband’s education, as well as ideal family size and residence area, are associated with intentions to have another child (Model 2). In a setting like Korea where male-breadwinner families are dominant and the labor market is highly gender-segmented, household income is primarily determined by men’s rather than women’s earnings. Thus men’s employment and education are more strongly associated with fertility intentions than women’s education. The lower intention to have another child among women with blue-collar jobs can be attributed to the large number of women who returned to the labor market after completing their childbearing. Leaving the labor market when marrying and becoming pregnant was common for women in Korea until recent years, and some of them re-entered the labor market to supplement household income after their children started school. Due to career discontinuity and gender-segmented labor market structure, however, their return to the labor market concentrates in low-skilled, low-paid, and low-status blue-collar or temporary jobs. Furthermore, the progression to second- or third-order births has become increasingly selective on economic stability (e.g., job security) in the last decades, whereas having a first child remains universal among married couples (Kim 2007, 2013; Yoo 2014). Finally, as could be expected, higher ideal family size and living in a rural area are associated with higher likelihood of wanting another child.

**Fig. 3:**
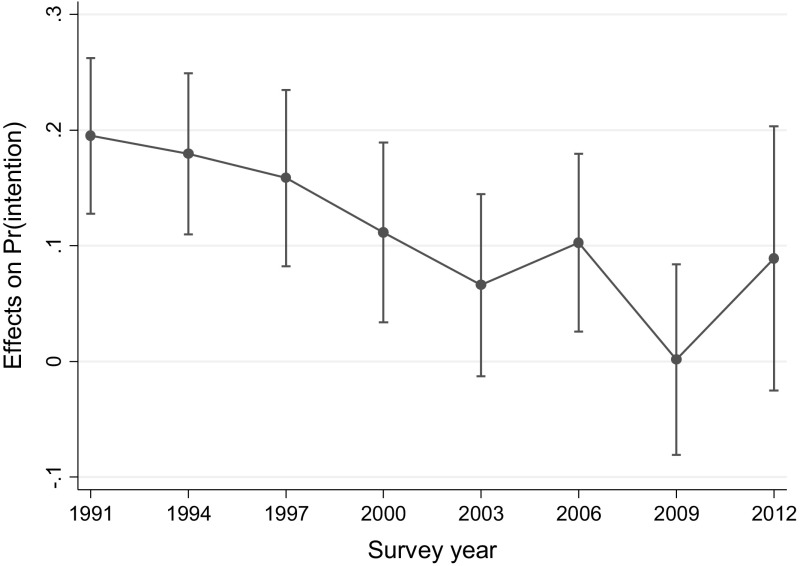
Conditional marginal effects of having a daughter on intention to have another child (ref: having a son) with 95 % CIs when other covariates are at means. *Note* The model controls for both men’s and women’s educational attainment and employment status, women’s age, residence area, ideal family size, and stated son preference only among married women (15–44) with a child (see the text for details). Reference is women with a son *Source* National Survey on Fertility, Family Health and Welfare, various years (1991–2012)

Conditional marginal effects of having a daughter and interactions with survey year were calculated and are displayed in Fig. 3. Marginal effects of having a daughter (relative to having a son) on fertility intentions clearly show a downward trend in son preference. The probability of intending to have another child for women with a daughter is 19.5 percentage points higher than those with a son in 1991, but gradually declines over time when other covariates are held at means. The marginal effect of having a daughter on intention to have another child becomes insignificant in all years starting from 2003 (at *p* > .05), except in 2006. Women with a daughter want to have a second child 8.6 percentage points more than women with a son in 2012, but the difference was not statistically significant (*p* < .05).

Changes in individual characteristics do not fully account for the yearly declines in son preference over time. In comparing Y-standardized coefficients between Model 1 and 2, adding individual characteristics into the model slightly attenuated the main effect of having a daughter (0.42 → 0.32). The standardized coefficients for survey year–daughter interaction terms are also smaller in all years in Model 2, indicating that the weakening of sex-composition effects on fertility intentions in the 1990s and 2000s was at least partly attributable to individual-level changes in women’s social position. However, the year–daughter interactions remain statistically significant in Model 2. The fact that not all of the decline in son preference is accounted for by controlling for individual characteristics suggests that widely shared societal changes also played a role in this trend.

**Fig. 4:**
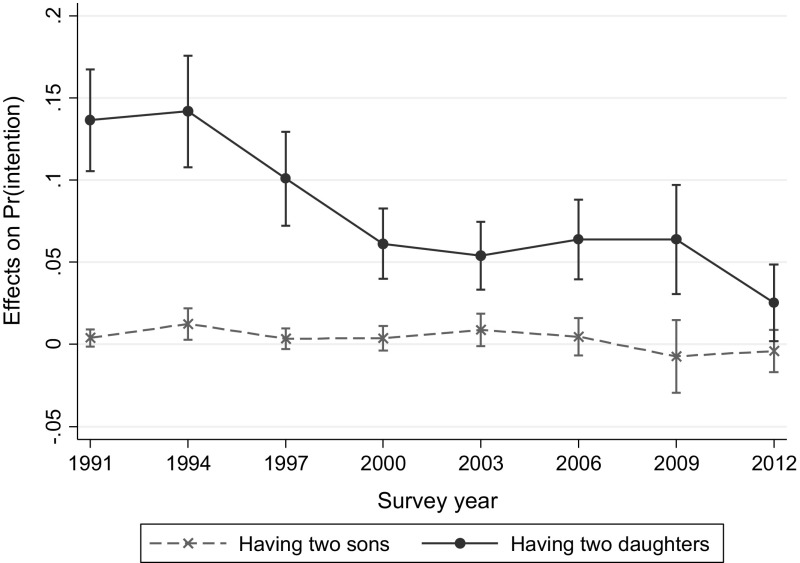
Conditional marginal effects of having same-sex children on intention to have another child with 95 % CIs when other covariates are at means. *Note* The model controls for both men’s and women’s educational attainment and employment status, women’s age, residence area, ideal family size, and stated son preference only among married women (15–44) with two children (see the text for details). Reference is women with the balanced-sex (a son + a daughter) children. *Source* National Survey on Fertility, Family Health and Welfare, various years (1991–2012)

Table 4 shows the same multivariate models estimated for women with two children. Again, calculated marginal effects of having same-sex children from Model 2 are shown in graphic form (Fig. 4). Here, the reference category for sex composition of children is women with one son and one daughter; women with two daughters, and women with two sons are compared with this reference. As in the models for women with one child, in Model 1 women with daughters only are more likely to want another child than women with one daughter and one son. The main effect term is large and positive: in 1991, the reference survey year, women with no sons had 16 times (exp(2.79) = 16.28) higher odds of wanting a third child than those with a daughter and a son. The interaction terms with survey year are all negative and statistically significant, indicating that this difference decreased over time. The total effect of having two daughters on fertility intention gradually diminished and eventually became insignificant in 2012 (2.79–2.44 = .34, ns) in the reduced model.

**Table 4:** Logistic regression of intentions to have another child on sex of children and other covariates, women with two children. *Source* National Survey on Fertility, Family Health and Welfare, various years (1991–2012)

	Model I			Model II		
	*b*	RSE	bStdY	*b*	RSE	bStdY
Sex of two children (ref: mixed-sex)						
Two sons	0.30	0.26	0.15	0.44	0.27	0.18
Two daughters	2.79	0.19***	1.44	3.13	0.21***	1.31
Interaction w/survey (ref: mixed-sex* 1991)						
Two sons * 1994	0.25	0.32	0.13	0.16	0.34	0.07
Two sons * 1997	−0.13	0.35	−0.07	−0.16	0.36	−0.07
Two sons * 2000	−0.04	0.37	−0.02	−0.15	0.38	−0.06
Two sons * 2003	−0.04	0.33	−0.02	−0.01	0.35	−0.01
Two sons * 2006	−0.11	0.33	−0.06	−0.24	0.35	−0.10
Two sons * 2009	−0.34	0.36	−0.18	−0.62	0.40	−0.26
Two sons * 2012	−0.53	0.36	−0.28	−0.59	0.37	−0.25
Two daughters * 1994	−0.66	0.26*	−0.34	−0.68	0.27*	−0.28
Two daughters * 1997	−0.84	0.27**	−0.43	−0.72	0.29*	−0.30
Two daughters * 2000	−1.09	0.29***	−0.57	−1.25	0.31***	−0.52
Two daughters * 2003	−1.55	0.27***	−0.80	−1.66	0.29***	−0.69
Two daughters * 2006	−1.60	0.27***	−0.83	−1.72	0.29***	−0.72
Two daughters * 2009	−1.89	0.28***	−0.98	−2.18	0.30***	−0.91
Two daughters * 2012	−2.44	0.33***	−1.26	−2.47	0.34***	−1.03
Stated son preference (ref: none)						
Better have a son				0.67	0.09***	0.28
Must have a son				1.07	0.10***	0.45
Ideal family size (ref: two children)						
Don’t know				−0.48	0.55	−0.20
Less than two children				−0.50	0.19*	−0.21
More than three children				1.34	0.07***	0.56
Residence (ref: metro)						
Small city				−0.06	0.08	−0.02
Rural				0.16	0.08^†^	0.07
Woman’s education: (ref: ≤HS comp.)						
Some college				0.19	0.12	0.08
Bachelor or higher				0.05	0.12	0.02
Woman’s employment: (ref: not working)						
White-collar job				−0.15	0.11	−0.06
Blue-collar job				−0.12	0.09	−0.05
Husband’s education: (ref: ≤HS comp.)						
Some college				0.00	0.11	0.00
Bachelor or higher				0.06	0.11	0.03
Husband’s employment: (ref: not working)						
White-collar job				−0.15	0.22	−0.06
Blue-collar job				−0.09	0.21	−0.04
Woman’s age (ref: <25)						
25–29				−0.27	0.28	−0.11
30–34				−0.98	0.28***	−0.41
35–39				−2.22	0.28***	−0.93
40–44				−3.54	0.31***	−1.47
Survey year (ref: 1991)						
1994	0.65	0.20**	0.34	0.78	0.21***	0.33
1997	0.23	0.22	0.12	0.43	0.22^†^	0.18
2000	0.04	0.23	0.02	0.49	0.24*	0.21
2003	0.36	0.21^†^	0.19	0.87	0.22***	0.36
2006	0.51	0.21*	0.27	1.15	0.23***	0.48
2009	0.92	0.22***	0.48	1.87	0.23***	0.78
2012	0.48	0.22*	0.25	1.39	0.24***	0.58
Constant	−3.6456	0.16***		−3.46	0.38***	
Log pseudolikelihood	−4685.00			−3803.70		
*N* (unweighted)	22,008			22,008		

Sample includes women with two children with no missing data on independent or dependent variables. Normalized weight was used. RSE denotes Robust Standard Errors and bStdY indicates Y-standardized coefficient

^†^ *p* < .10; * *p* < .05; ** *p* < .01; *** *p* < .001

The result is robust whether or not we include reported son preference and individual characteristics, except that the total size effect of having two daughters in 2012 became significant (3.13–2.47 = 0.66, *p* < .05). When individual characteristics are added in Model 2, the standardized coefficients for sex composition of previous children do not differ substantially from previous models in Table 4. The standardized coefficients for the survey year–sex composition interaction terms are attenuated, but the downward trend in the effect size of having two daughters remains robust. Although individual characteristics, such as both spouses’ education and employment, and residence area, contributed to the decline in the effect of having two daughters on the likelihood of having a third child, changes in these characteristics were not the main driver of the decline. Rather, compared to the results for second birth intentions above, these individual characteristics appear to have played a smaller role in changes in third birth intentions, probably because third births became increasingly rare in the country.

Compared to women with one son and one daughter, having two sons is not associated with intentions to have another child. The positive direction of this association may imply a desire for a mixed-sex composition of children, suggesting that women with no daughters are more likely to want another child than women with one son and one daughter. However, the interaction terms with year are negative, though again not reaching statistical significance. Thus, any hint at mixed-sex preference noticeable in the earlier survey years is no longer present by the end of the period under observation.

Reported son preference is also strongly and positively associated with wanting another child. Both reported son preference and the sex composition of children maintain independent associations with fertility intentions. It also suggests that having son preference, whether it is strong or moderate, is one of the important determinants for wanting another child among women with two children. The effects of other covariates show similar directions as shown in Table 3.

Figure 4 presents conditional marginal effects of having same-sex children on intentions to have another child when other covariates are at means. Compared to having one son and one daughter, having two sons does not show any difference in fertility intentions for a third child from the beginning, while having two daughters is associated with significantly higher intentions through the study period. Note that both upper and lower limits of 95 % CI for having two daughters (solid line) are higher than zero, which indicates a statistically significant difference in effects from having one son and one daughter. Despite a clear downward trend, intentions to have a third child significantly differed by sex composition of existing children until 2012 (*p* < .05 when all covariates are at means).

Interestingly, the main effect coefficients for survey year are also positive and statistically significant. For women with one son and one daughter, the reference category, the likelihood of wanting another child increased over the period of 1991–2012 in the models in Table 4, consistent with the descriptive statistics (Table 2). It is not clear why the intention for a third child increased in this period despite the continued decline in overall birth rates. Possible explanations may lie in changes of the demographics of women with two children during the observation period. We do not explore this further because changes in fertility intentions have been explicitly analyzed in other studies (Kim 2007, 2013).

## Discussion and Conclusions

Korea’s SRB peaked at extremely high levels in the early 1990s but declined to about normal (105 males per 100 females) in the late 2000s. The Korean case is often presented as a success story for the decline of unbalanced SRBs. However, changes in the underlying attitudes that accompanied this behavioral change are not well understood. To examine this process, we compared trends in both population-level (SRLB) and individual-level (reported son preference, fertility intentions) measures of son preference and analyzed how the relationship between the intention to have another child and the sex of existing children changed over time.

Consistent with reported trends in SRB, we find substantial evidence of declining son preference in Korea. The SRLB declined along with the SRB; women are less likely to report son preference when directly asked about the importance of having a son; and the association of the sex of existing children with intentions for future fertility weakened over time. Despite these changes, however, son preference attitudes persist. Around 41 % of women in the most recent survey reported that it was necessary or desirable to have at least one son, and intentions to have another child continue to vary depending on sex composition of existing children, in a direction consistent with son preference.

The SRLB rapidly declined and eventually reached near normal levels of the SRB by 2012, but that also suggests that a gender-based “stopping rule” was still being used in recent years. It also evidences that preferential attitudes favoring sons over daughters still exist among women in their middle of reproductive span despite the remarkable decline in son preference. These attitudes can have important consequences for differences in the sibship size and gender composition—under a gender-based stopping rule, girls will have more siblings than boys, on average, and are more likely to be older siblings. In some contexts, child’s sex is also predictive of parental divorce and living arrangements, but this association has not yet been tested in Korea (Dahl and Moretti 2008). Thus, even as SRB normalizes, son preference can continue to have demographic consequences in low-fertility contexts.

We find that the effect of son preference on intentions for further childbearing differs by birth order. Fertility intentions to have a second child became dissociated from sex of first child in recent years, which is consistent with prior studies that found no son preference in low-fertility contexts (Andersson et al. 2006; Pollard and Morgan 2002). However, son preference still influences intentions for further childbearing among women with two children in Korea. At parity two, women with two daughters are more likely to want another child than are women with two sons. The stronger intention to have another child among women with two daughters, compared to those with two sons, is consistent and robust over the observed period (1991–2012). Although the effect sizes of the sex composition considerably decreased during that period, the difference in fertility intentions between women with two daughters and those with one son and one daughter remains substantial and at levels far higher than in other countries (cf. Duthé et al. 2012; Pollard and Morgan 2002).

We do not find any evidence of preference for mixed-sex children. Although the desire for mixed-sex children is common in many developed countries, it has not been found among Korean women who have sons only (Arnold 1985; Larsen et al. 1998). In the contemporary Korean context, where more couples have only one child, preference for mixed-sex children may be seen as harder to achieve than son preference because it requires giving birth to at least two children.

What factors brought Korea’s SRB to a normal level in recent years? Recent research commonly points to modernization and urbanization as major causes for such normalization (Chung and Das Gupta 2007; Das Gupta 2010). Our results suggest that individual-level changes in urban residence, ideal family size, or both partners’ education and employment offer just a partial explanation for the declines in the association between the sex composition of existing children and intentions for future fertility. Individual-level change explained a substantial portion of the declining difference in fertility intentions between women with only one daughter and women with only one son. For women with two children, changes in son preference are distributed across the whole population, pointing to the importance of broader social and institutional change and transformation of the family system. Improvements in gender inequity, such as a declining gender-gap in wage, can be one of the reasons (e.g., Lee 2013). Despite Korea’s patrilocal tradition, living with the husband’s parents has also become increasingly less common in the recent decades, and thus, traditional reasons for son preference, such as family succession, ancestral rites, and old age security, have faded away rapidly.

These changes may be attributable to either period- or cohort-based processes or, most likely, a combination of both. The technological changes that made sex-selective abortion possible, thus facilitating the rise of SRB, were period changes, as were the legal changes that prohibited sex-selective abortion and the policy changes that encouraged more gender-egalitarian family systems. However, attitudes about family size and desirable sex composition of children are often formed and solidified in adolescence and young adulthood, and thus change in attitudes is typically a cohort-driven process.

Our study has some limitations. First, our data are limited to married women. However, this limitation has minimal effects given that childbearing mostly occurs in marital union in Korea; more than 97 % of births were to married mothers in 2012. Second, our analysis is based on cross-sectional data. Hence, our findings stand on association rather than causal relationship, and we could not account for possible selectivity in examining attitudes and intentions among women who already had two children. Third, we use women’s intention to have another child, rather than actual behavior, as our outcome. Although fertility intentions are closely related to fertility behavior, the extent to which intentions are translated into behavior varies across diverse contexts. It is possible that intentions related to son preference are more (or less) likely to be carried out than nongender specific intentions; we are not aware of any research that tests for this possibility. Lastly, our analysis could not account for sex-selective abortion, which is a major mechanism for translating son preference into behavior.

These limitations notwithstanding, our study provides a unique insight into changes in son preference in a transition from low fertility to lowest-low fertility. The rapid changes in SRBs over the past two decades in Korea were caused not so much by changes in preferences, as by changes in the cultural, economic, and institutional environment. Underlying preferences for sons persist in Korea, but the realization of these preferences has changed. It remains to be seen how and to what extent, if at all, these preferences will manifest themselves in the future. In a post-transitonal context with very low fertility, sex preferences for children, whether they are toward sons, daughters, or a mix of both sexes, might actually help raise fertility slightly, assuming that sex-selective abortion is no longer a desirable option (cf. Bongaarts 2001). Moreover, in a society where small family size has established itself as a strong social norm, attenuated son preference might evolve from selection of sex composition of children to gendered types and amount of investment in children, such as education and time allocation for daughters vs. sons. For example, parents may spend more time and resources on raising a first child if that child is a boy than a girl (e.g., Choi and Hwang 2015). Finally, as assisted reproductive technologies become more widely used in Korea, it is also possible that sex selection of embryos may have an impact on SRB in the future. In sum, our findings suggest that the disappearance of unbalanced SRB does not necessarily mean the emergence of sex indifference, and the disappearance of son preference is not an inevitable consequence of very low fertility.

## Appendix

See Tables 5, 6, 7, and 8.

**Table 5:** The mean age of married women aged 15–44 by the number of children and survey. *Source* National Survey on Fertility, Family Health and Welfare, various years (1991–2012)

Year	Number of children				Total
	No child	One	Two	Three	
1991	26.6	30.0	34.2	38.6	33.6
1994	27.7	30.4	34.8	38.8	33.8
1997	28.3	31.6	35.8	38.4	34.6
2000	28.7	32.2	36.6	37.9	35.0
2003	30.2	33.6	37.2	38.6	36.1
2006	30.5	33.9	37.4	38.5	36.1
2009	31.6	34.1	37.9	38.7	36.5
2012	32.0	34.8	38.0	38.6	36.5
Total	29.4	32.5	36.4	38.5	35.2

All figures were weighted

**Table 6:** Logistic regression of intentions to have another child in women of age 25–34, with one child. *Source* National Survey on Fertility, Family Health and Welfare, various years (1991–2012)

	Model I			Model II		
	*b*	RSE	bStdY	*b*	RSE	bStdY
Sex of first child (ref: a son)						
A daughter	0.67	0.15***	0.36	0.77	0.17***	0.37
Interaction w/survey (ref: a son * 1991)						
A daughter * 1994	0.01	0.24	0.01	0.05	0.26	0.02
A daughter * 1997	−0.31	0.23	−0.17	−0.27	0.26	−0.13
A daughter * 2000	−0.39	0.23†	−0.21	−0.39	0.25	−0.19
A daughter * 2003	−0.57	0.23*	−0.31	−0.64	0.26*	−0.31
A daughter * 2006	−0.43	0.23†	−0.23	−0.34	0.25	−0.17
A daughter * 2009	−0.93	0.27**	−0.50	−0.99	0.28**	−0.48
A daughter * 2012	−0.35	0.37	−0.19	−0.39	0.36	−0.19
Stated son preference (ref: none)						
Better have a son				0.44	0.08***	0.21
Must have a son				0.82	0.12***	0.40
Ideal family size (ref: two children)						
Don’t know				−0.76	0.37*	−0.37
Less than two children				−1.55	0.10***	−0.75
More than three children				0.40	0.11***	0.19
Residence (ref: metro)						
Small city				0.00	0.09	0.00
Rural				0.35	0.09***	0.17
Woman’s education: (ref: ≤HS comp.)						
Some college				0.01	0.13	0.01
Bachelor or higher				0.05	0.15	0.03
Woman’s employment: (ref: not working)						
White-collar job				−0.12	0.11	−0.06
Blue-collar job				−0.33	0.10**	−0.16
Husband’s education: (ref: ≤HS comp.)						
Some college				0.44	0.13**	0.21
Bachelor or higher				0.52	0.16**	0.25
Husband’s employment: (ref: not working)						
White-collar job				−0.03	0.26	−0.02
Blue-collar job				0.02	0.26	0.01
Woman’s age (ref: 25–29)						
30–34				−0.81	0.08***	−0.39
Survey year (ref: 1991)						
1994	0.44	0.15**	0.24	0.24	0.16	0.12
1997	0.16	0.15	0.09	−0.12	0.17	−0.06
2000	−0.03	0.14	−0.02	−0.30	0.17^†^	−0.14
2003	−0.09	0.15	−0.05	−0.26	0.17	−0.13
2006	−0.23	0.15	−0.12	−0.61	0.17***	−0.30
2009	0.47	0.18*	0.26	0.38	0.20^†^	0.18
2012	−0.12	0.27	−0.06	−0.14	0.27	−0.07
Constant	0.50	0.09***		0.76	0.28**	
Log pseudo−likelihood	−3434.37			−3032.94		
*N* (unweighted)	5328			5328		

Sample includes women with one child with no missing data on independent or dependent variables. Normalized weight was used. RSE denotes Robust Standard Errors and bStdY indicates Y-standardized coefficient

^†^ *p* < .10; * *p* < .05; ** *p* < .01; *** *p* < .001

**Table 7:** Logistic regression of intentions to have another child among in women of age 25–34, with two children. *Source* National Survey on Fertility, Family Health and Welfare, various years (1991–2012)

	Model I			Model II		
	*b*	RSE	bStdY	*b*	RSE	bStdY
Sex of two children (ref: mixed-sex)						
Two sons	0.41	0.29	0.21	0.46	0.30	0.21
Two daughters	2.69	0.22***	1.37	3.13	0.23***	1.42
Interaction w/survey (ref: mixed-sex* 1991)						
Two sons * 1994	0.13	0.37	0.07	0.15	0.39	0.07
Two sons * 1997	−0.16	0.39	−0.08	−0.10	0.41	−0.05
Two sons * 2000	−0.04	0.43	−0.02	−0.02	0.44	−0.01
Two sons * 2003	−0.02	0.39	−0.01	0.03	0.41	0.02
Two sons * 2006	−0.11	0.40	−0.05	−0.23	0.42	−0.10
Two sons * 2009	−0.63	0.43	−0.32	−0.89	0.46^†^	−0.40
Two sons * 2012	−0.59	0.44	−0.30	−0.45	0.46	−0.20
Two daughters * 1994	−0.53	0.30^†^	−0.27	−0.58	0.31^†^	−0.27
Two daughters * 1997	−0.77	0.32*	−0.39	−0.80	0.33*	−0.36
Two daughters * 2000	−1.33	0.35***	−0.68	−1.36	0.36***	−0.62
Two daughters * 2003	−1.56	0.32***	−0.79	−1.59	0.34***	−0.72
Two daughters * 2006	−1.58	0.33***	−0.80	−1.71	0.36***	−0.78
Two daughters * 2009	−1.88	0.37***	−0.96	−2.21	0.38***	−1.00
Two daughters * 2012	−2.59	0.42***	−1.32	−2.48	0.41***	−1.13
Stated son preference (ref: none)						
Better have a son				0.78	0.10***	0.36
Must have a son				1.28	0.12***	0.58
Ideal family size (ref: two children)						
Don’t know				−0.40	0.64	−0.18
Less than two children				−0.63	0.24**	−0.29
More than three children				1.30	0.08***	0.59
Residence (ref: metro)						
Small city				0.05	0.10	0.02
Rural				0.17	0.10^†^	0.08
Woman’s education: (ref: ≤HS comp.)						
Some college				0.16	0.14	0.07
Bachelor or higher				0.09	0.14	0.04
Woman’s employment: (ref: not working)						
White-collar job				−0.02	0.14	−0.01
Blue-collar job				−0.13	0.10	−0.06
Husband’s education: (ref: ≤HS comp.)						
Some college				0.07	0.13	0.03
Bachelor or higher				0.04	0.13	0.02
Husband’s employment: (ref: not working)						
White-collar job				−0.14	0.27	−0.06
Blue-collar job				−0.07	0.26	−0.03
Woman’s age (ref: 25−29)						
30−34				−0.72	0.09***	−0.33
Survey year (ref: 1991)						
1994	0.75	0.23**	0.38	0.79	0.24**	0.36
1997	0.56	0.25*	0.28	0.57	0.25*	0.26
2000	0.53	0.27†	0.27	0.62	0.28*	0.28
2003	0.89	0.24***	0.45	0.93	0.25***	0.42
2006	1.08	0.25***	0.55	1.29	0.27***	0.59
2009	1.59	0.27***	0.81	1.86	0.29***	0.84
2012	1.26	0.27***	0.64	1.43	0.29***	0.65
Constant	−3.21	0.18***		−3.95	0.34***	
Log pseudolikelihood	−2673.02			−2362.40		
*N* (unweighted)	7698			7698		

Sample includes women with two children with no missing data on independent or dependent variables. Normalized weight was used. RSE denotes Robust Standard Errors and bStdY indicates Y-standardized coefficient

^†^ *p* < .10; * *p* < .05; ** *p* < .01; *** *p* < .001

**Table 8:** Proportion of sex combination among women with one child and two children. *Source* National Survey on Fertility, Family Health and Welfare, various years (1991–2012)

	One child			Two children			
	Dau.	Son	All	2 dau.	1 dau. + 1 son	2 sons	All
SRB 105	48.8	51.2		23.8	50.0	26.2	
SRB 107	48.3	51.7		23.3	49.9	26.7	
1991	42.9	57.1	100.0 (1275)	16.0	54.0	30.1	100.0 (2847)
1994	42.8	57.2	100.0 (1112)	17.5	55.1	27.4	100.0 (2615)
1997	42.7	57.3	100.0 (1069)	15.5	55.9	28.6	100.0 (2998)
2000	43.0	57.0	100.0 (1100)	17.1	55.5	27.4	100.0 (2761)
2003	41.9	58.2	100.0 (1026)	16.6	55.2	28.2	100.0 (3161)
2006	43.9	56.1	100.0 (1037)	18.3	54.5	27.3	100.0 (2751)
2009	44.3	55.7	100.0 (972)	20.6	52.6	26.8	100.0 (2325)
2012	49.5	50.5	100.0 (1139)	19.6	54.4	26.0	100.0 (2643)
Total	44.0	56.0	100.0 (8730)	17.5	54.7	27.8	100.0 (22,101)

The SRB 105 and 107 represent the proportion of sex composition when we assume 105 males and 107 males per 100 females as natural level of SRB, respectively. All percentages were weighted, and unweighted *N* placed in the parenthesis
